# Meta-topologies define distinct anatomical classes of brain tumours linked to histology and survival

**DOI:** 10.1093/braincomms/fcac336

**Published:** 2022-12-22

**Authors:** Julius M Kernbach, Daniel Delev, Georg Neuloh, Hans Clusmann, Danilo Bzdok, Simon B Eickhoff, Victor E Staartjes, Flavio Vasella, Michael Weller, Luca Regli, Carlo Serra, Niklaus Krayenbühl, Kevin Akeret

**Affiliations:** Neurosurgical Artificial Intelligence Laboratory Aachen (NAILA), RWTH Aachen University Hospital, Pauwelsstrasse 30, 52074 Aachen, Germany; Department of Neurosurgery, Faculty of Medicine, RWTH Aachen University, Pauwelsstrasse 30, 52074 Aachen, Germany; Center for Integrated Oncology, Düsseldorf (CIO ABCD), Universities Aachen, Bonn, Cologne, Germany; Neurosurgical Artificial Intelligence Laboratory Aachen (NAILA), RWTH Aachen University Hospital, Pauwelsstrasse 30, 52074 Aachen, Germany; Department of Neurosurgery, Faculty of Medicine, RWTH Aachen University, Pauwelsstrasse 30, 52074 Aachen, Germany; Center for Integrated Oncology, Düsseldorf (CIO ABCD), Universities Aachen, Bonn, Cologne, Germany; Department of Neurosurgery, Faculty of Medicine, RWTH Aachen University, Pauwelsstrasse 30, 52074 Aachen, Germany; Center for Integrated Oncology, Düsseldorf (CIO ABCD), Universities Aachen, Bonn, Cologne, Germany; Department of Neurosurgery, Faculty of Medicine, RWTH Aachen University, Pauwelsstrasse 30, 52074 Aachen, Germany; Center for Integrated Oncology, Düsseldorf (CIO ABCD), Universities Aachen, Bonn, Cologne, Germany; Department of Biomedical Engineering, McConnell Brain Imaging Centre, Montreal Neurological Institute, Faculty of Medicine, School of Computer Science, McGill University, 845 Sherbrooke St W, Montreal, Quebec H3A 0G4, Canada; Mila—Quebec Artificial Intelligence Institute, 6666 Rue Saint-Urbain, Montreal, Quebec H2S 3H1, Canada; Institute of Neuroscience and Medicine (INM-7), Research Centre Jülich, Wilhelm Johnen Strasse, 52428 Jülich, Germany; Institute of Systems Neuroscience, Medical Faculty, Heinrich Heine University Düsseldorf, Moorenstrasse 5, 40225 Düsseldorf, Germany; Department of Neurosurgery, Clinical Neuroscience Center, University Hospital and University of Zurich, Frauenklinikstrasse 10, 8091 Zurich, Switzerland; Department of Neurosurgery, Clinical Neuroscience Center, University Hospital and University of Zurich, Frauenklinikstrasse 10, 8091 Zurich, Switzerland; Department of Neurosurgery, Clinical Neuroscience Center, University Hospital and University of Zurich, Frauenklinikstrasse 10, 8091 Zurich, Switzerland; Department of Neurosurgery, Clinical Neuroscience Center, University Hospital and University of Zurich, Frauenklinikstrasse 10, 8091 Zurich, Switzerland; Department of Neurosurgery, Clinical Neuroscience Center, University Hospital and University of Zurich, Frauenklinikstrasse 10, 8091 Zurich, Switzerland; Department of Neurosurgery, Clinical Neuroscience Center, University Hospital and University of Zurich, Frauenklinikstrasse 10, 8091 Zurich, Switzerland; Division of Pediatric Neurosurgery, University Children's Hospital, Steinwiesstrasse 75, 8032 Zurich, Switzerland; Department of Neurosurgery, Clinical Neuroscience Center, University Hospital and University of Zurich, Frauenklinikstrasse 10, 8091 Zurich, Switzerland

**Keywords:** anatomy, artificial intelligence, machine learning, glioma, metastases

## Abstract

The current World Health Organization classification integrates histological and molecular features of brain tumours. The aim of this study was to identify generalizable topological patterns with the potential to add an anatomical dimension to the classification of brain tumours. We applied non-negative matrix factorization as an unsupervised pattern discovery strategy to the fine-grained topographic tumour profiles of 936 patients with neuroepithelial tumours and brain metastases. From the anatomical features alone, this machine learning algorithm enabled the extraction of latent topological tumour patterns, termed *meta-topologies*. The optimal part-based representation was automatically determined in 10 000 split-half iterations. We further characterized each meta-topology’s unique histopathologic profile and survival probability, thus linking important biological and clinical information to the underlying anatomical patterns. In neuroepithelial tumours, six meta-topologies were extracted, each detailing a transpallial pattern with distinct parenchymal and ventricular compositions. We identified one infratentorial, one allopallial, three neopallial (parieto-occipital, frontal, temporal) and one unisegmental meta-topology. Each meta-topology mapped to distinct histopathologic and molecular profiles. The unisegmental meta-topology showed the strongest anatomical–clinical link demonstrating a survival advantage in histologically identical tumours. Brain metastases separated to an infra- and supratentorial meta-topology with anatomical patterns highlighting their affinity to the cortico-subcortical boundary of arterial watershed areas.Using a novel data-driven approach, we identified generalizable topological patterns in both neuroepithelial tumours and brain metastases. Differences in the histopathologic profiles and prognosis of these anatomical tumour classes provide insights into the heterogeneity of tumour biology and might add to personalized clinical decision-making.

## Teaser

Kernbach et al. present a novel data-led framework capitalizing on non-negative matrix factorization to deconvolute generalizable topological patterns in brain tumours (meta-topologies). The meta-topologies identified in primary brain tumours map to distinct histopathologic, molecular and clinical findings.

## Graphical Abstract

**Graphical abstract:**
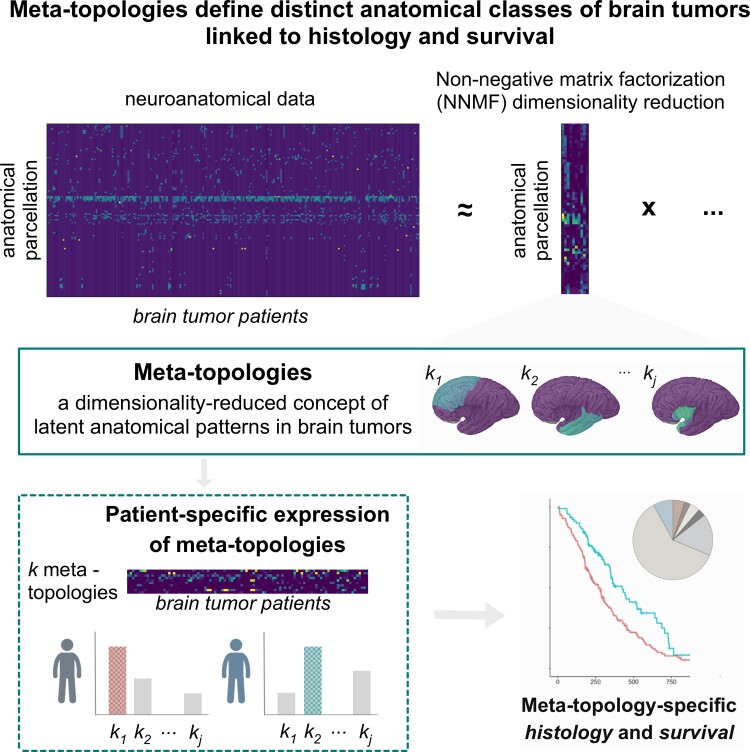

## Introduction

Each year, over 300 000 people worldwide are diagnosed with brain tumours, which cause more than 200 000 deaths and 7 600 000 disability-adjusted life years.^1^ In recent decades, major advances in the histologic and molecular profiling of brain tumours have been achieved and implemented into classification systems and diagnostic guidelines.^2,3^ The most recent WHO Classification of Tumours of the Central Nervous System further strengthens this integration of molecular and histological parameters.^4,5^ The anatomical phenotype of brain tumours, however, is of minor relevance in this classification. Previous reports indicate an association between tumour location and biological tumour signature.^6-9^ We propose a unified data-driven framework tailored to identify generalizable topological patterns, which may enhance our understanding and the classification of brain tumours.^8,9^

Specific anatomical patterns are found in numerous neurological diseases. Neurodegenerative disorders differ in their atrophy patterns,^10^ or autoinflammatory diseases preferentially affect distinct CNS structures.^11^ The molecular mechanisms behind this selective vulnerability of the brain, referred to as pathoclisis,^12^ remain largely elusive. In brain tumours, descriptions of topographic prevalence and relative spatial density also implicate differences in the anatomical phenotype.^8,9^ Defining anatomical classes of brain tumours requires the identification of topological relationships that are consistent across patients. Topographic analyses can provide a description of the involvement of individual anatomical structures. However, analyzing each neuroanatomical location as a single unit deters intuitive interpretation by omitting interaction effects between spatially adjacent or distant areas.

In contrast, studies on topology aim to incorporate the relative positions of the individual anatomical components to each other.^13^ We propose a novel framework tailored to identify generalizable patterns in brain tumour topology using non-negative matrix factorization (NNMF).^14^ Factorization methods, including NNMF, are often applied in the analyses of genomic signatures across various cancer types^15-17^ and provide unique advantages for the purpose of the present study. The inherent non-negative constraint of the applied algorithm enables an intuitive and direct interpretation of the derived patterns. In contrast, alternative dimensionality reduction tools such as principal component analysis would hurt intuitive interpretation of any distributed effects by recovering patterns through incomprehensible combinations of positive and negative cancellations of the extracted low-dimensional patterns.^14^ Further, NNMF is considered a sum-of-parts approach. It purposefully can appreciate the mutual functional dependence between individual neuroanatomical locations and enables an interpretation on a topological level.

In the present computational study, we design an unsupervised data-led approach using machine learning to identify an optimal factorization of latent neuroanatomical meta-patterns in brain tumours that we henceforth call *meta-topologies*. First, we extract a low-dimensional embedding from the fine-grained neuroanatomical distributions using unsupervised pattern discovery. Second, we assess the subject-specific expression of the derived tumour configurations for their histopathologic identity and prognostic relevance. By introducing brain tumour meta-topologies, we intend to supplement our biological understanding and the individual profiling of brain tumours to inform individualized treatment decisions and assist in tailored therapy customized to the single patient.^18,19^

## Materials and methods

This study was approved by the ethical review board of the Canton of Zurich, Switzerland (KEK ZH 01120). Reporting of results is in accordance with the STROBE statement.^20^

### Data source

Topographic tumour profiles were obtained from a previously published and openly available single-centre cohort of *n* = 1000 consecutive patients with newly diagnosed brain tumours (https://doi.org/10.5281/zenodo.5457402).^9^ The eligibility criteria comprised (i) first diagnosis with consecutive histopathologic confirmation of a neuroepithelial tumour or brain metastases; (ii) no pretreatment or previous cranial surgery; (iii) intraparenchymal encephalic tumour location; (iv) availability of preoperative MRI data. For a detailed description of the acquired demographic (sex, age), clinical (Karnofsky Performance Status, type of surgery, chemotherapy, radiotherapy), radiological (presurgical 3-tesla Skyra VD13 MRI, Siemens Healthcare, Erlangen, Germany, with a 24- or 32-channel receive coil) and histopathological (histological and molecular characterization of MIB-1, 1p19q, IDH, MGMT promoter methylation) data, we refer to the protocol of the original cohort paper.^9^ Each patient’s topographic tumour profile was based on a standardized whole-brain parcellation protocol^21^ and contained 120 anatomical annotations. We excluded 44 patients with primary central nervous system lymphoma and 20 tumours with indistinct gyral patterns for the analyses in this study.

### Data-led deconvolution of hidden tumour meta-topologies

We sought to explore coherent topological anatomical patterns that may be hidden in the rich spatial descriptions of the topographic phenotypes (Supplementary Fig. 1). We capitalized on NNMF as a lossy multivariate pattern discovery strategy.^14^ This unsupervised machine learning algorithm can identify the form and patient-specific combination of latent topological patterns that together compose the individual neuroanatomical tumour phenotypes. These derived sum-of-parts representations are henceforth called *meta-topologies*. More formally, NNMF achieves a low-rank approximation of the data *V*, with *V* reflecting the 120 topographic summaries, with dimensions of *m* × *n* (*m* = number of anatomical items, *n* = number of patients), by partitioning the interindividual variation in anatomical items into a basic matrix *W* of *k* part-based factor representations. The matrix of the latent factor loadings *H* indicated how relevant each emerging meta-topology was to describe an individual patient’s tumour phenotype. Accordingly, *W* and *H* carried *m* × *k* and *k* × *n* dimensions, respectively. Given by *V = WH*, the latent factorization decomposed the actual tumour phenotype in a particular patient into a part-based representation.

In contrast to alternative dimensionality reduction methods, NNMF provided at least two significant advantages for the goal of the investigation. First, the nature of the neuroanatomical data encoded as non-negative values and the build-in non-negativity constraint of the NNMF algorithm allows for intuitive and meaningful interpretation. Alternative matrix factorization algorithms, such as principal component analysis (Supplementary Fig. 2), typically involve complex cancellations between positive and negative numbers, which lack intuitive meaning and would have hurt the neurobiological interpretation. Second, NNMF is considered a parts-based learning approach^14^ and only allows strictly additive combinations of meta-topology contributions. That way, NNMF is compatible with the intuitive notion of combining non-negative parts, e.g. expressed meta-topologies, to form the individual tumour phenotype. In contrast, other classical clustering approaches, e.g. independent component analysis (ICA), recover independent holistic representations of the data. However, the independence assumption made by ICA is ill-suited for learning parts-based representations.

### Optimal factorization based on quantitative model evaluation

To find the optimal parts-based representation, we capitalized on robustness measures and the ability to generalize to new populations. We applied a data-driven out-of-sample evaluation strategy to determine the most robust and generalizable representations of tumour meta-topologies across 10 000 bootstrapped split-half iterations. We quantitatively assessed the resulting factorizations for (a) generalizability by measuring the increase in out-of-sample reconstruction error (RE)^22^ and (b) stability using the adjusted Rand Index (aRI).^23,24^ The optimal factorization should be reflected in both a lower increase in out-of-sample RE and a higher aRI in the majority of the iterations.

We quantified the out-of-sample RE by projecting the data of one half onto the latent dimensions from the other half. The RE represents the absolute difference between the reconstructed and original matrix. Accordingly, the increase in out-of-sample RE demonstrates how much worse the data matrix is reconstructed by the basis matrix obtained from the model-unseen sample compared to the basis matrix recovered from the within-sample split. A lower increase in out-of-sample RE compared with the within-sample RE hence indicates better generalizability.

Stability was assessed using the aRI. As a modified version of the Rand Index, the aRI is stricter and allows for improved discrimination.^23,24^ The aRI is adjusted for chance; that is, the aRI penalizes for the placement of two data points from different true clusters into the same cluster. The aRI is ensured to have values close to 0 for random labelling and yields a value between −1 and +1, with negative values when the index is less than the expected index. In this study, the aRI was used to measure the correspondence between the factorizations derived from the two split samples based on the assignment of the anatomical items to the part-based meta-topologies. Higher values of aRI indicate better correspondence between the two factorizations derived in separate split-half realizations, and a value of 1 represents an identical assignment.

We selected the most generalizable and robust factorization with rank *k* when the mean increase in out-of-sample RE of the all 10 000 split-half realizations was minimized, while the mean aRI was maximized.

### Statistical analysis

Chi-square test was applied for categorical variables (with continuity correction) and ANOVA for continous data. The Kaplan Meier method was used to estimate survival probabilities of tumour meta-topologies, and the log-rank test with Bonferroni-Holm correction for multiple testing was applied for pairwise comparisons.^25^ Multivariable survival analysis was performed using the Cox proportional hazards regression model.^26,27^ Unadjusted models were compared to models with adjustment for histopathologic tumour subtype. Given the exploratory nature of this study, the results were interpreted based on the level of evidence without the definition of a level of statistical significance: *P* < 0.001: very strong evidence; *P* < 0.01: strong evidence; *P* < 0.05 evidence; *P* < 0.1 weak evidence; *P* > 0.1: no evidence.^28^

### Data and code availability

Data analyses were conducted in Python 3.8.5 (IPython 7.21.0) and R 4.0.0 (RStudio 1.3.1093) environments. Full datasets and codes are available online (https://doi.org/10.5281/zenodo.5515356).

## Results

Detailed demographic, histopathologic, and clinical cohort characteristics are provided in Supplementary Table 1. We quantitatively assessed the most robust and generalizable NNMF factorization for neuroepithelial tumours and brain metastases separately by combining the maximized mean aRI and the out-of-sample RE with the least relative increase (Supplementary Fig. 3). We extracted the optimal factorization as *k* = 6 meta-topologies in neuroepithelial tumours (Supplementary Fig. 3A) and *k* = 2 in brain metastases (Supplementary Fig. 3B).

### Meta-topologies in neuroepithelial tumours

**Figure 1:**
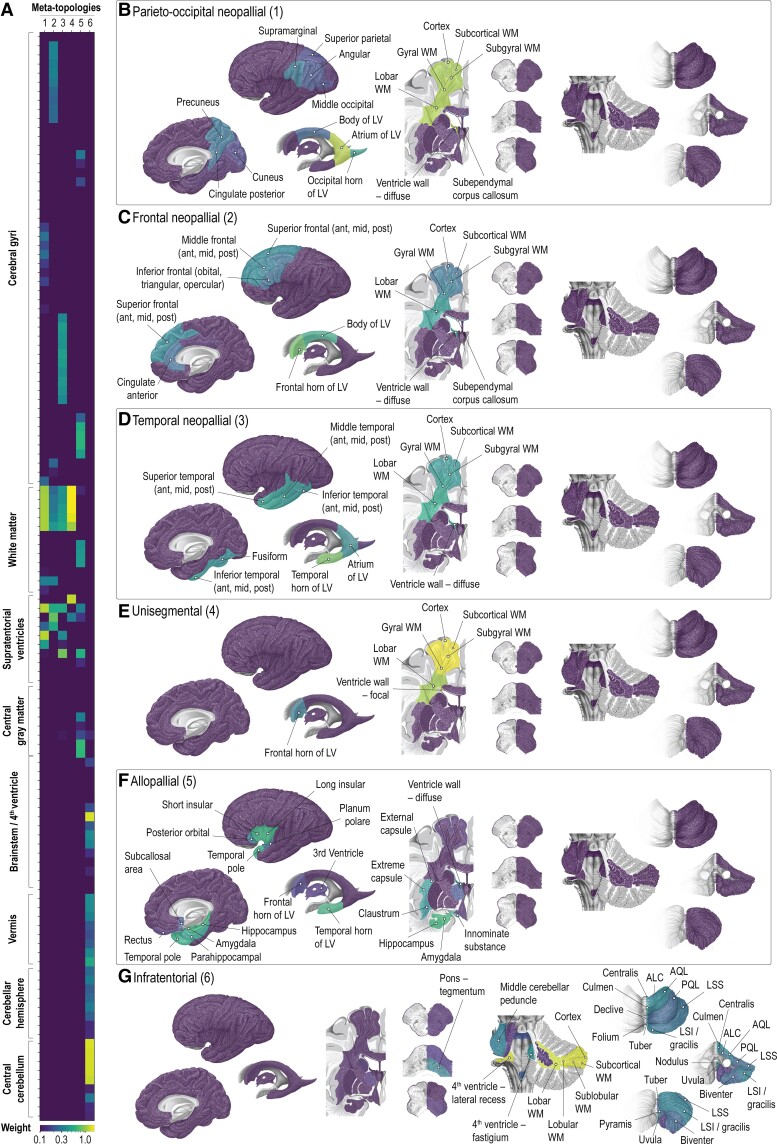
**Meta-topologies in neuroepithelial tumours.** (**A**). Meta-topologies in neuroepithelial tumours with their respective anatomical configurations. A detailed description of the anatomical distributions is provided in Supplementary Fig. 4. (**B-G).** Spatial visualizations of three neopallial (1: parieto-occipital; 2: frontal; 3: temporal), unisegmental (4), allopallial (5) and infratentorial (6) meta-topologies. The anatomical items with the highest differential weight are labelled. ALC, ala lobuli centralis; Ant, anterior third; AQL, anterior quadrangular lobule; LSI, inferior semilunar lobule; LSS, superior semilunar lobule; LV, lateral ventricle; mid, middle third; post, posterior third; PQL, posterior quadrangular lobule; WM, white matter.

We identified six meta-topologies in neuroepithelial tumours. Each meta-topology showed a distinct composition of anatomical items (Fig. 1A, Supplementary Fig. 4A, Supplementary Table 2) and level of gyrality (Supplementary Table 3) that informed post-hoc naming. The composition of the meta-topologies did not relevantly change when corrected for lesion volume or hemispheric lateralization (Supplementary Fig. 5 and 6). Meta-topologies 1–5 predominantly reflected supratentorial anatomy. Infratentorial structures were captured in meta-topology 6. Distinct combinations of gyral, ventricular and radial tumour anatomy characterized the unique constellations of meta-topologies 1–5: we identified three meta-topologies with neopallial mapping (parieto-occipital, frontal, temporal), one with predominantly allopallial enrichment and one meta-topology lacking a gyral pattern (unisegmental).

#### Parieto-occipital neopallial neuroepithelial tumours (meta-topology 1, Fig. 1B)

Most relevant anatomical items included the atrium of the lateral ventricle (loading weight 1.19), diffuse involvement of the wall of the lateral ventricle (1.17), all cerebral white matter sectors (lobar and gyral 1.11, subgyral 1.10, subcortical 1.09) and the cerebral cortex (1.07). In addition, meta-topology 1 mapped to the occipital horn and the body of the lateral ventricle, the subependymal corpus callosum, and gyri of the parieto-occipital neopallium (supramarginal, precuneus, angular, middle occipital, superior parietal, cuneus). Neuroepithelial tumours characterized primarily by the parieto-occipital neopallial meta-topology 1 (*n* = 141) were multigyral in 46.1% (*n* = 65), unigyral in 49.6% (*n* = 70) and affected no cerebral gyrus in 4.3% (*n* = 6).

#### Frontal neopallial neuroepithelial tumours (meta-topology 2, Fig. 1C)

The frontal horn of the lateral ventricle featured the highest loading weight (0.82), followed by the body of the lateral ventricle (0.52), diffuse involvement of the wall of the lateral ventricle (0.52) and the cerebral lobar white matter sector (0.43). Meta-topology 2 was located in the gyral, subgyral, and subcortical white matter sectors, the cerebral cortex, and gyri of the fronto-medial neopallium (superior, middle, and inferior frontal gyri, anterior cingulate gyrus). Neuroepithelial tumours characterized by the frontal neopallial meta-topology 2 (*n* = 115), showed a multigyral involvement in 57.4% (*n* = 66), unigyral in 27.8% (*n* = 32), and no gyral involvement in 14.8% (*n* = 17).

#### Temporal neopallial neuroepithelial tumours (meta-topology 3, Fig. 1D)

Meta-topology 3 was uniquely defined by the temporal horn of the lateral ventricle (0.86), diffuse involvement of the wall of the lateral ventricle (0.53), and the cerebral lobar white matter sector (0.52). Meta-topology 3 also mapped to the other cerebral radial sectors (gyral, subgyral, subcortical), the cerebral cortex, and temporal neopallial gyri (superior, middle, and inferior temporal, fusiform). The atrium of the lateral ventricle was also affected, yet to a lesser extent than the temporal horn. Neuroepithelial tumours mapping predominantly on the temporal neopallial meta-topology 3 (*n* = 69) were multigyral in 56.5% (*n* = 39), unigyral in 39.1% (*n* = 27) and involved no gyrus in 4.3% (*n* = 3).

#### *Unisegmental neuroepithelial* tumour*s* (meta-topology 4, Fig. 1E)

Meta-topology 4 represented supratentorial neuroepithelial tumours mapping to the cerebral cortex (1.58) and all cerebral white matter sectors (subcortical 1.57, subgyral 1.54, gyral 1.41, lobar 1.07). Ventricular involvement was primarily limited to focal contact to the wall of the lateral ventricle (1.25), specifically in the frontal horn of the lateral ventricle (0.30). Gyral constellations showed no relevant contribution to meta-topology 4. Notably, neuroepithelial tumours belonging to meta-topology 4 (*n* = 179) were dominated by a pronounced unigyral character (82.1%, *n* = 147; 17.9% (*n* = 32) multigyral).

#### *Allopallial neuroepithelial* tumour*s* (meta-topology 5, Fig. 1F)

Meta-topology 5 was dominated by the amygdala (0.65), the hippocampus (0.62), long (0.61) and short (0.60) insular gyri, the temporal horn of the lateral ventricle (0.57), temporal pole (0.56), parahippocampal gyrus (0.47), innominate substance (0.44), as well as extreme (0.43) and external (0.41) capsules. Meta-topology 5 additionally mapped to the claustrum, posterior orbital gyrus, subcallosal area, planum polare, and thalamus, as well as the frontal horn of the lateral ventricle and the third ventricle. Diffuse involvement of the wall of the lateral ventricle added more relevantly to the constellation of the meta-topology than a focal ventricular contact. Neuroepithelial tumours mapping predominantly on the allopallial meta-topology 5 (*n* = 70) were multigyral in 55.7% (*n* = 39), unigyral in 12.9% (*n* = 9) and involved no gyrus in 31.4% (*n* = 22).

#### Infratentorial neuroepithelial tumours (meta-topology 6, Fig. 1G)

The last meta-topology was determined by infratentorial structures: the cerebellar cortex (1.37), the lateral recess of the fourth ventricle (1.35), as well as the cerebellar white matter sectors (subcortical 1.34, sublobular 1.34, lobular 1.33, lobar 1.27). Both the vermian and the hemispheric lobules contributed to the neuroanatomical constellation of meta-topology 6. Of the neuroepithelial tumours matching best on meta-topology 6 (*n* = 72), 98% (*n* = 71) did not involve a cerebral gyrus. There was only one tumour (1.4%) with multigyral anatomy.

Collectively, all identified meta-topologies in neuroepithelial tumours involved distinct ventricular segments and shared a characteristic transpallial pattern. Each supratentorial meta-topology presented a distinct gyral pattern except for meta-topology 4, which was predominantly unigyral in nature. The unigyral character of meta-topology 4 was uniquely associated with a strong contribution of a focal contact to the ventricle wall, while all other supratentorial meta-topologies depicted a diffuse ventricular involvement.

### Meta-topologies in brain metastases

**Figure 2:**
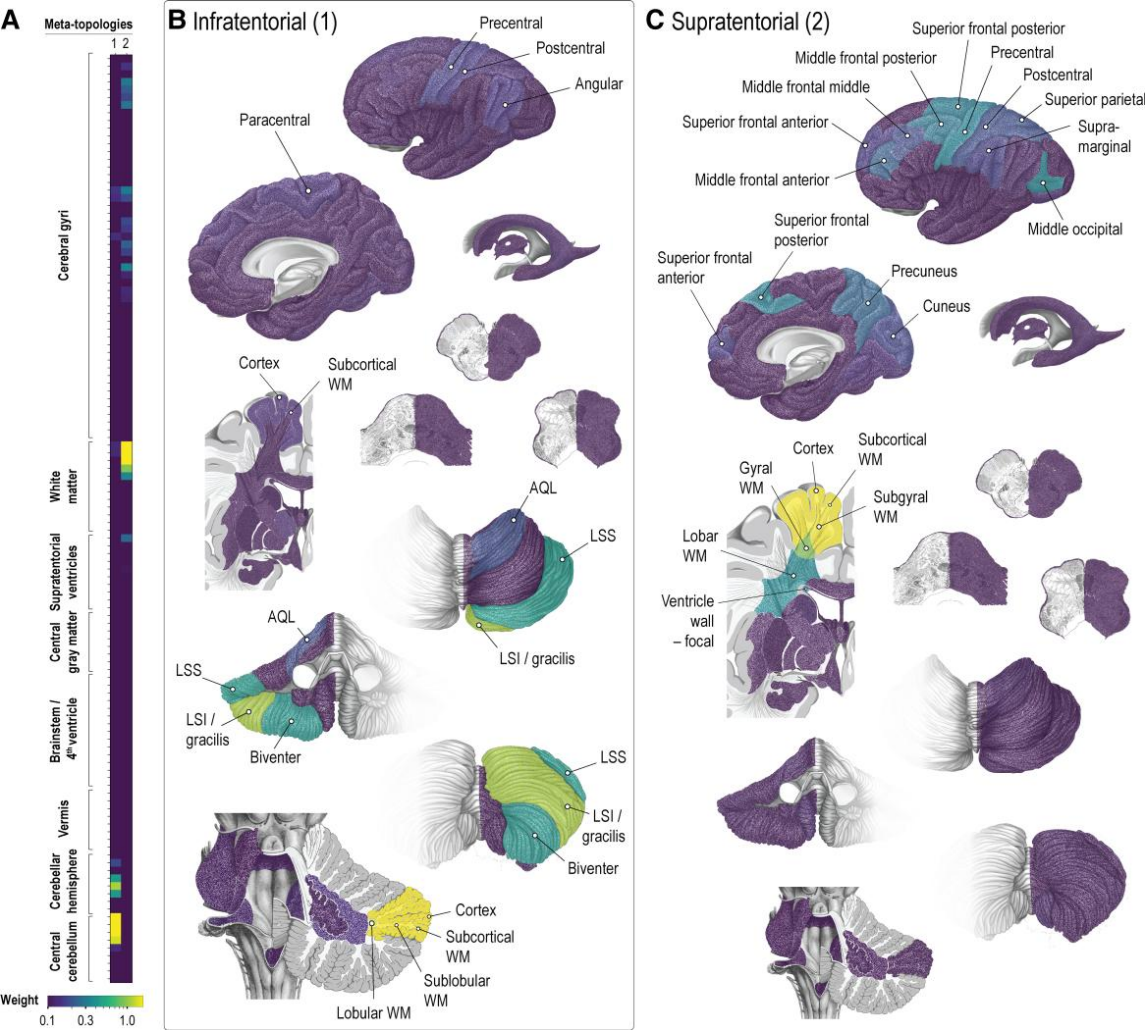
**Meta-topologies in brain metastases.** (**A**) Meta-topologies in brain metastases with their respective anatomical configurations. A detailed description of the anatomical distributions is provided in Supplementary Fig. 4. (**B and C)** Spatial visualization of the infra- (1) and supratentorial (2) meta-topologies. The anatomical items with the highest differential weight are labelled. AQL, anterior quadrangular lobule; LSI, inferior semilunar lobule; LSS, superior semilunar lobule; LV, lateral ventricle; PQL, posterior quadrangular lobule; WM, white matter.

We quantitatively identified two meta-topologies in brain metastases (Fig. 2A, Supplementary Fig. 4B and Table 4). A separation between infratentorial (meta-topology 1) and supratentorial (meta-topology 2) topological patterns emerged.

#### Infratentorial brain metastases (meta-topology 1, Fig. 2B)

The topological pattern was dominated by the cerebellar cortex (2.18) and the superficial cerebellar white matter sectors (subcortical 2.17, sublobular 1.94, lobular 1.49). In addition, meta-topology 1 mapped strongly to the inferior semilunar/gracilis lobule (1.24) and, although less pronounced, to the superior semilunar (0.47), biventer (0.45) and anterior quadrangular (0.19) lobules. There was also discrete enrichment in specific supratentorial items, such as the precentral, postcentral, paracentral, angular gyri, and the cerebral cortex and cerebral subcortical white matter sector.

#### Supratentorial brain metastases (meta-topology 2, Fig. 2C)

Meta-topology 2 was again determined by superficial structures, namely the cerebral cortex (2.83) and cerebral subcortical white matter sector (2.83), followed by the cerebral subgyral white matter sector (2.30). Weaker enrichment was seen in the cerebral gyral (1.03) and lobar (0.37) white matter sectors. On a gyral level, we found the strongest factor contribution in the middle occipital (0.36), precentral (0.34), posterior third of the superior frontal (0.32), and posterior third of the middle frontal (0.30) gyri. Meta-topology 2 also mapped to the anterior third of the superior frontal, anterior two-thirds of the middle frontal, the postcentral and supramarginal gyri, the superior parietal lobule, precuneus and cuneus. We observed focal contact to the wall of the lateral ventricle (0.26) but no diffuse involvement.

In summary, both meta-topologies in brain metastases showed a strong affinity to the cortex (cerebellar or cerebral) and superficial white matter sectors (especially subcortical). The observed cerebellar lobular and cerebral gyral patterns were consistent with the expected anatomical locations of arterial border zones, i.e. watershed areas. The gyral pattern seen in meta-topology 2 was weakly represented in meta-topology 1, likely due to synchronous supra- and infratentorial metastases. Unlike in neuroepithelial tumours, ventricular segments and deep white matter sectors were not associated with meta-topologies in brain metastases.

### Brain tumour meta-topologies map to distinct histopathologic and molecular profiles

**Figure 3:**
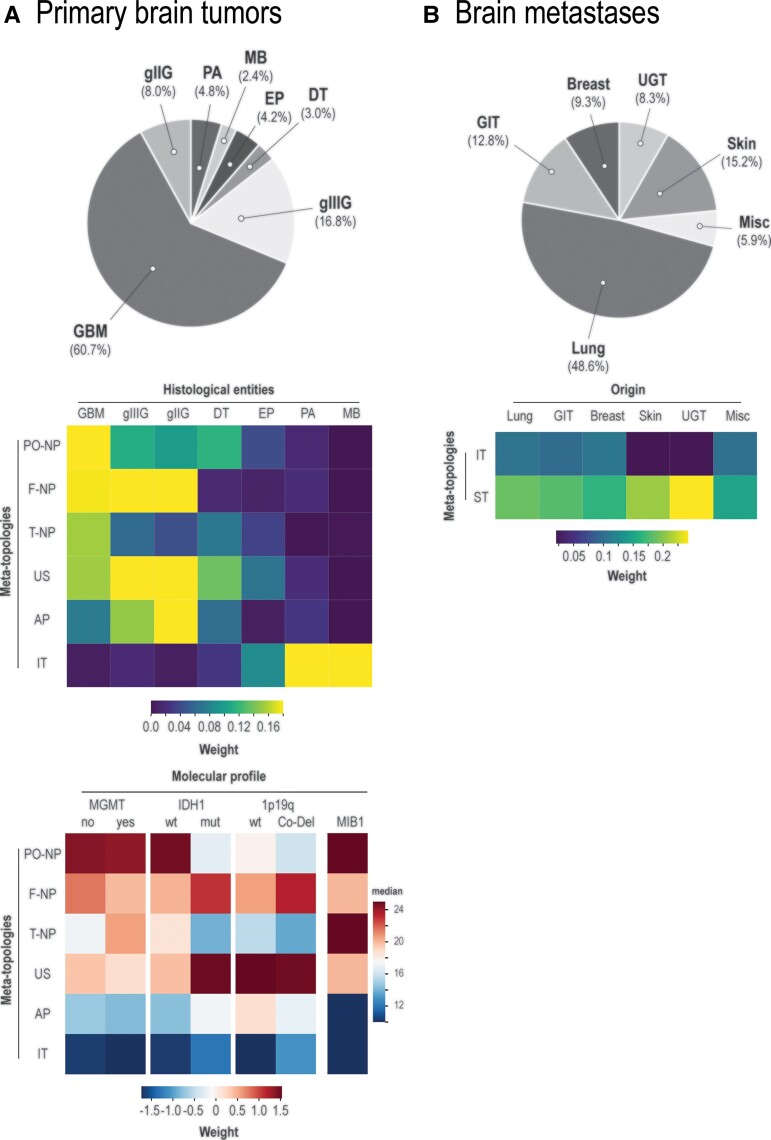
**Histopathologic and molecular profiling of brain tumour meta-topologies.** (**A**) *Top:* Relative frequency of different histologic entities in the neuroepithelial tumour cohort. *Upper matrix:* Mean expression of each meta-topology across neuroepithelial tumour entities. *Lower matrix:* Summarized mean expression of each meta-topological molecular profiling based on mutational status (mean weight) and stratified median MIB-1/Ki-67 proliferation index. 1p19q, 1p19q co-deletion; AP, allopallial; DT, developmental tumour; EP, ependymoma; F-NP, frontal neopallial; g2G, WHO Grade 2 gliomas; g3G, WHO Grade 3 glioma; g4G, WHO Grade 4 gliomas (glioblastoma/IDH-mutant Grade 4 astrocytomas); IDH1, isocitrate dehydrogenase (IDH) 1 mutation; IT, infratentorial; MB, medulloblastoma; MGMT, O(6)-methylguanine-DNA methyltransferase (MGMT) promoter methylation; MIB1, MIB-1/Ki-67 proliferation index; PA, pilocytic astrocytoma, PO-NP, parieto-occipital neopallial; T-NP, temporal neopallial; US, unisegmental. (**B**) *Top:* Relative frequency of different brain metastases subtypes (depending on organ of origin). *Matrix:* Meta-topology-specific enrichment (weight) of brain metastases subtypes. GIT, gastrointestinal tract (mouth, tonsil, parotid, esophagus, stomach, gallbladder, pancreas, colorectal cancer); IT, infratentorial, Misc, miscellaneous (cancer of unknown primary, adrenal, leukaemia, sarcoma, mesothelial, thyroid); ST, supratentorial; UGT, urogenital tract (kidney, bladder; ovary, tube, uterus; testes, prostate).

The dominant histopathologic entity in neuroepithelial tumours was WHO Grade 4 glioma (60.7%), followed by gliomas of WHO Grade 3 (16.8%) and Grade 2 (8.0%) (Fig. 3A). The histopathologic entities spread differently to the identified meta-topologies in neuroepithelial tumours. WHO grades 2–4 gliomas, and developmental tumours mapped primarily to supratentorial meta-topologies. Ependymoma appeared in both supra- and infratentorial meta-topologies, while pilocytic astrocytoma and medulloblastoma were almost exclusively associated with the infratentorial pattern (meta-topology 6). WHO Grade 4 gliomas mapped to all neopallial [parieto-occipital (1), frontal (2), and temporal (3)], and the unisegmental (4) meta-topology but showed distinct molecular patterns. A high proliferation index (MIB-1 monoclonal antibodies) was associated predominantly with meta-topologies 1 (parieto-occipital neopallial) and 3 (temporal neopallial). Tumours mapping to meta-topology 3 (temporal neopallial) showed a preference for MGMT promoter methylation. IDH-1 mutations mapped strongly to meta-topology 4 (unisegmental) and, although less prominently, to meta-topology 2 (frontal neopallial) and 5 (allopallial). IDH-1 wild-type status was prominent in meta-topology 1 (parieto-occipital neopallial) and, to a lesser extent, in meta-topology 3 (temporal neopallial). 1p19q co-deletion status was associated with meta-topology 2 (frontal neopallial). WHO Grade 3 gliomas mapped prominently to meta-topologies 2 and 4, i.e. the frontal neopallial and unisegmental types. Their contribution to meta-topology 5 (allopallial) and meta-topology 1 (parieto-occipital neopallial) was intermediate. WHO Grade 2 glioma showed a similar pattern of factor contribution with dominance in meta-topologies 2 (frontal neopallial), 4 (unisegmental), and 5 (allopallial) and intermediate weight in meta-topology 1 (parieto-occipital neopallial).

Brain metastases most frequently arose from lung cancer (48.6%), melanoma (15.2%), or gastrointestinal cancer (12.8%) (Fig. 3B). Brain metastases from lung, gastrointestinal or breast cancer contributed relevantly to both meta-topologies. However, there was a pronounced preference of brain metastases from melanoma and urogenital cancer to meta-topology 2, i.e. the supratentorial type.

### Brain tumour meta-topologies uncover survival differences

**Figure 4:**
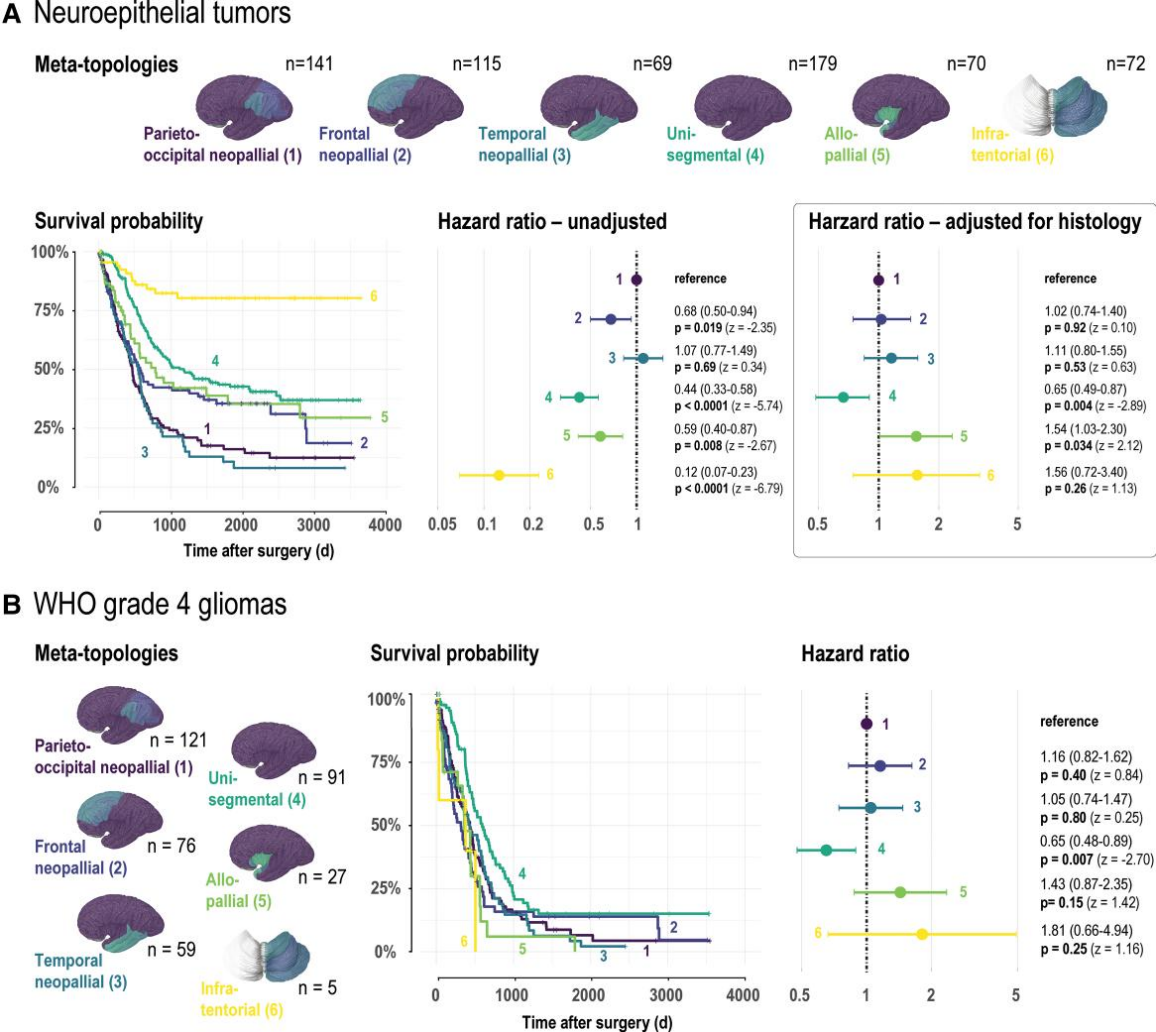
**Meta-topologies and patient survival in primary brain tumours.** (**A**) Survival in patients with neuroepithelial tumours stratified by the corresponding meta-topology with the highest weight (brain visualizations). *Left:* Kaplan–Meier curves. Risk tables and censoring events are given in Supplementary Fig. 7A. *Right:* Forest plots providing meta-topology-specific hazard ratios with 95% confidence intervals and *P*-values (Wald statistic z) based on unadjusted and histology-adjusted Cox proportional hazards analyses. *Abbreviations:* d, days. **(B)** Survival in patients with WHO Grade 4 gliomas stratified by the corresponding meta-topology with the highest weight (brain visualizations). *Left:* Kaplan–Meier curves. Risk tables and censoring events are given in Supplementary Fig. 7A. *Right:* Forest plots providing meta-topology-specific hazard ratios with 95% confidence intervals and *P*-values (Wald statistic z) based on Cox proportional hazard analysis.

**Figure 5:**
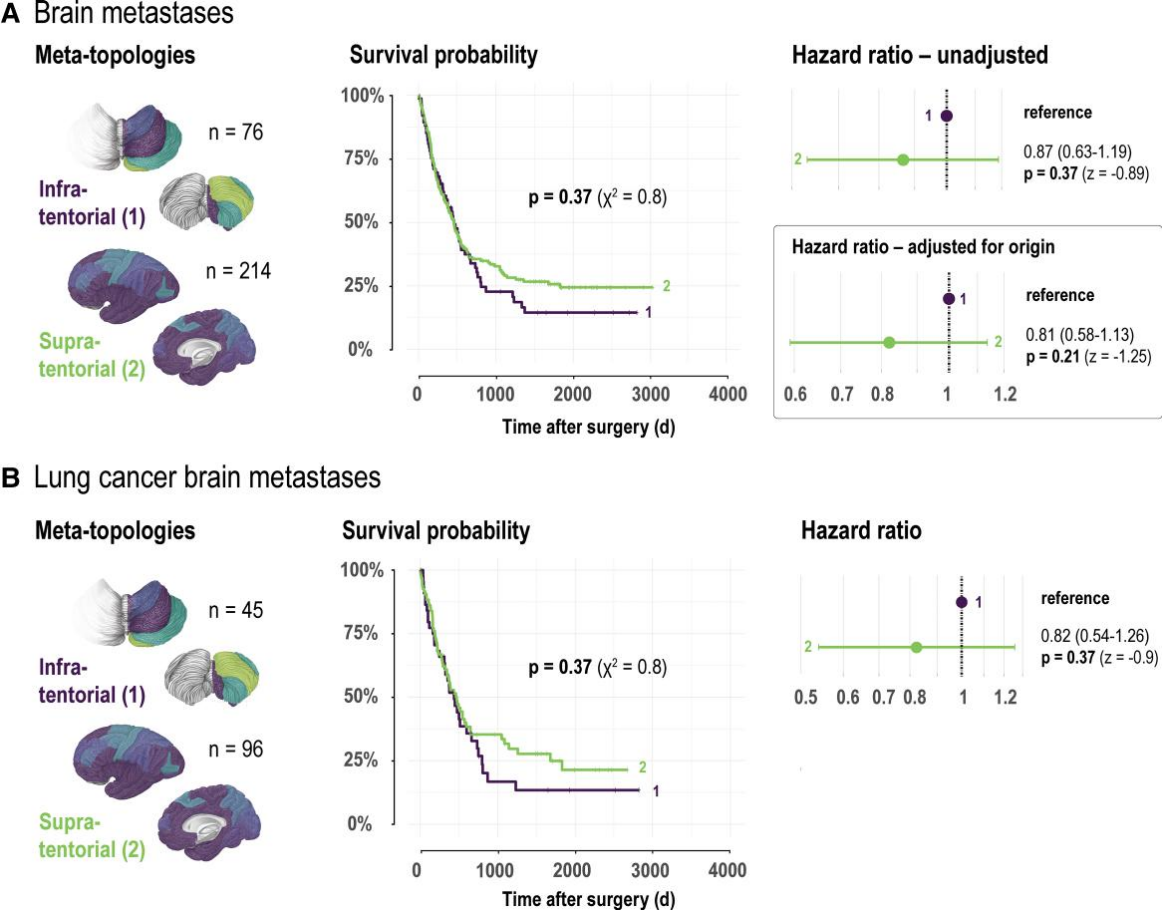
**Meta-topologies and patient survival in brain metastases.** (**A**) Survival in patients with brain metastases stratified by the corresponding meta-topology with the highest weight (brain visualizations). *Left:* Kaplan–Meier curves. Risk tables and censoring events are given in Supplementary Fig. 7B. The *P*-value is based on the log-rank test (chi-square statistic χ^2^). *Right:* Forest plots providing meta-topology-specific hazard ratios with 95% confidence intervals and *P*-values (Wald statistic z) based on unadjusted and origin-adjusted Cox proportional hazards analyses. (**B**) Survival in patients with lung cancer brain metastases stratified by the corresponding meta-topology with the highest weight (brain visualizations). *Left:* Kaplan–Meier curves. Risk tables and censoring events are given in Supplementary Fig. 7B. The *P*-value is based on the log-rank test (chi-square statistic χ^2^). *Right:* Forest plots providing meta-topology-specific hazard ratios with 95% confidence intervals and *P*-values (Wald statistic z) based on Cox proportional hazard analysis.

For survival analysis, patients were individually assigned to their highest expressed meta-topology (Tables 1 and 2). In neuroepithelial tumours, meta-topology 6 (infratentorial) showed the highest survival probability (hazard ratio 0.12, 95% confidence interval: 0.07 to 0.23, *P* < 0.0001; Fig. 4A, Supplementary Table 5A), thus supporting the NNMF results, which predominantly mapped ependymoma and pilocytic astrocytoma to this meta-topology. Poorest prognosis was seen in patients with tumours of meta-topologies 1 (parieto-occipital neopallial, reference for Cox proportional hazards analysis) or 3 (temporal neopallial, hazard ratio 1.07, 95% confidence interval: 0.77–1.49, *P* = 0.69) consistent with the histopathologic findings as both meta-topologies specifically mapped to WHO Grade 4 gliomas. The analysis emphasizes the histopathologic– and anatomical–clinical link uncovered by the unsupervised and data-led analysis strategy. To capture hidden information beyond the histopathologic characteristics that might be directly linked to the clinical course, we adjusted for the histopathologic entities and showed that patients with tumours classified as meta-topology 4 (unisegmental) demonstrated a survival advantage (hazard ratio 0.65, 95% confidence interval: 0.49–0.87, *P* = 0.004). The survival analysis in WHO Grade 4 gliomas (Fig. 4B, Supplementary Table 5B) provided further evidence that patient with tumours matching to meta-topology 4 (unisegmental) show a better overall survival (hazard ratio 0.65, 95% confidence interval: 0.48 to 0.89, *P* = 0.007), underlining the anatomical–clinical link within histopathologically identical tumours. In contrast, brain metastases showed no evidence for a difference in survival between patients with assigned to the infra- or supratentorial meta-topology across all analyses (brain metastases overall unadjusted or adjusted for origin, and lung cancer brain metastases) (Fig. 5A and B).

**Table 1:** Cohort characteristics in neuroepithelial tumours stratified by dominant meta-topology

Meta-topology	Overall	1	2	3	4	5	6	*P*-value
Neuroepithelial tumours overall								
***n***	646	141	115	69	179	70	72	
**Sex (male)**	402 (62.2)	88 (62.4)	64 (55.7)	48 (69.6)	119 (66.5)	39 (55.7)	44 (61.1)	0.271
**Age (years)**	50.1 (21.6)	59.6 (15.7)	54.0 (18.1)	57.7 (18.9)	49.4 (18.5)	50.4 (21.0)	19.1 (18.6)	<0.001
**Karnofsky Performance Status**	80.0 (12.6)	78.2 (11.5)	75.8 (14.6)	78.7 (12.5)	84.8 (10.5)	79.5 (12.1)	80.4 (14.0)	<0.001
**Modified Rankin Scale**	1.7 (1.0)	1.9 (0.9)	2.0 (1.0)	1.9 (1.0)	1.3 (0.8)	1.7 (0.9)	1.7 (1.0)	<0.001
**Resection (vs. biopsy)**	510 (78.9)	99 (70.2)	75 (65.2)	60 (87.0)	171 (95.5)	44 (62.9)	61 (84.7)	<0.001
**Chemotherapy**	428 (69.1)	100 (74.6)	71 (64.5)	50 (73.5)	135 (78.0)	40 (60.6)	32 (47.1)	<0.001
**Radiotherapy**	473 (76.2)	114 (84.4)	81 (73.6)	56 (82.4)	144 (82.8)	44 (66.7)	34 (50.0)	<0.001
**MIB1**	25.0 (20.6)	28.0 (18.0)	24.2 (18.2)	29.6 (21.4)	26.5 (22.1)	16.3 (17.0)	21.4 (24.3)	0.001
**1p19q co-deletion**	43 (31.4)	3 (21.4)	12 (52.2)	0 (0.0)	25 (34.7)	3 (13.6)	0 (0.0)	0.041
**IDH1 mutation** ^a^	88 (19.6)	8 (7.5)	17 (19.5)	1 (2.0)	53 (37.6)	9 (20.5)	0 (0.0)	<0.001
**MGMT promoter methylation**	95 (38.6)	28 (37.3)	18 (37.5)	17 (47.2)	24 (38.1)	7 (36.8)	1 (20.0)	0.854
**WHO Grade 4 gliomas**								
***n***	401	121	76	59	91	27	5	
**Sex (male)**	244 (64.4)	77 (63.6)	45 (59.2)	42 (71.2)	62 (68.1)	14 (51.9)	4 (80.0)	0.410
**Age (years)**	60.8 (14.2)	62.7 (12.3)	60.3 (14.7)	62.4 (13.7)	58.8 (15.4)	62.2 (8.6)	33.4 (25.1)	<0.001
**Karnofsky Performance Status**	76.9 (13.2)	77.4 (12.0)	72.0 (14.7)	77.9 (12.8)	81.2 (11.4)	74.8 (12.2)	60.0 (27.1)	<0.001
**Modified Rankin Scale**	1.9 (1.0)	2.0 (1.0)	2.2 (1.1)	1.9 (1.0)	1.6 (0.9)	2.1 (0.9)	3.2 (1.3)	<0.001
**Resection (vs. biopsy)**	285 (75.2)	85 (70.2)	47 (61.8)	50 (84.7)	87 (95.6)	15 (55.6)	1 (20.0)	<0.001
**Chemotherapy**	269 (73.7)	86 (74.1)	47 (64.4)	45 (77.6)	76 (85.4)	13 (52.0)	2 (50.0)	0.004
**Radiotherapy**	311 (85.0)	100 (85.5)	56 (76.7)	51 (87.9)	81 (91.0)	21 (84.0)	2 (50.0)	0.057
**MIB1**	32.5 (19.2)	30.6 (17.9)	30.0 (17.2)	32.8 (20.9)	36.8 (20.6)	31.7 (19.0)	40.6 (26.7)	0.183
**1p19q co-deletion**	2 (9.1)	0 (0.0)	1 (50.0)	0 (0.0)	1 (14.3)	0 (0.0)	0 (−)	NaN
**IDH1 mutation** ^a^	10 (3.4)	3 (3.2)	4 (6.5)	0 (0.0)	3 (4.1)	0 (0.0)	0 (0.0)	0.535
**MGMT promoter methylation**	82 (38.0)	28 (38.4)	14 (34.1)	17 (48.6)	17 (33.3)	6 (42.9)	0 (0.0)	0.584

The demographic, histopathologic, and clinical characteristics of the patients with neuroepithelial tumours overall and with WHO Grade 4 gliomas stratified by the dominant meta-topology. ^a^based on immunohistochemistry or PCR.

**Table 2:** Cohort characteristics in brain metastases stratified by dominant meta-topology

	Metastases overall				Lung cancer metastases			
Meta-topology	Overall	1	2	*P*-value	Overall	1	2	*P*-value
***n***	290	76	214		141	45	96	
**Gender (m)**	149 (51.4)	41 (53.9)	108 (50.5)	0.70	77 (54.6)	26 (57.8)	51 (53.1)	0.74
**Age (years)**	60.7 (11.9)	58.5 (11.9)	61.5 (11.8)	0.064	61.4 (9.9)	58.7 (9.7)	62.6 (9.8)	0.030
**Karnofsky Performance Status**	76.9 (11.6)	77.5 (10.5)	76.7 (12.0)	0.62	77.7 (11.0)	78.9 (10.3)	77.2 (11.4)	0.40
**Modified Rankin Scale**	2.0 (0.9)	1.9 (0.8)	2.0 (0.9)	0.53	1.9 (0.8)	1.8 (0.7)	1.9 (0.9)	0.52
**Resection (vs. biopsy)**	277 (95.5)	72 (94.7)	205 (95.8)	0.95	136 (96.5)	43 (95.6)	93 (96.9)	1.0
**Chemotherapy**	175 (62.3)	49 (68.1)	126 (60.3)	0.30	94 (69.1)	32 (72.7)	62 (67.4)	0.67
**Radiotherapy**	255 (89.2)	67 (90.5)	188 (88.7)	0.82	128 (92.1)	41 (93.2)	87 (91.6)	1.0

The demographic, histopathologic, and clinical characteristics of the patients with brain metastases overall and lung cancer brain metastases stratified by the dominant meta-topology.

## Discussion

We present a novel data-led approach using machine learning to explore generalizable topological patterns across different entities of brain tumours. Using a quantitative out-of-sample evaluation strategy, we extracted six distinct meta-topologies in neuroepithelial tumours and two meta-topologies in brain metastases in a cohort of 936 patients with fine-grained anatomical tumour annotations. The anatomical configuration of meta-topologies and their unique histopathologic profiles and prognoses provide insights into tumour biology and may enrich the current classification of brain tumours supporting more personalized clinical decision-making.

Previous studies on anatomical patterns in brain tumours have been restricted to descriptions of topographic prevalence. In contrast, the use of an NNMF approach allowed us to find latent topological patterns across different brain tumours and thus to infer possible segmental tumour behaviour in a purely data-driven fashion. The notion of latent meta-topologies with distinct histologic and molecular profiles in neuroepithelial tumours emphasizes the concept of pathoclisis encountered in various other neurological disorders.^10-12^ The interpretation of their spatial architecture could enhance our biological understanding of tumour origin and evolution. First, all meta-topologies in neuroepithelial tumours shared a transpallial character, i.e. all radial sectors between the cortex and ventricle were equally relevant. This was contrasted by the radial pattern in metastases with cerebellar (meta-topology 1) and cerebral (meta-topology 2) cortico-subcortical dominance and a ventriculopetal gradient. These findings are consistent with previous descriptions of the metastatic preference for the cortico-medullary boundary^29^ and a spatiotemporal behaviour of neuroepithelial tumours within ventriculo-cortical radial units.^8,9^ Second, except for meta-topology 4, the supratentorial meta-topologies identified in neuroepithelial tumours demonstrated sharply defined gyral patterns, implying a segmental parenchymal growth behaviour. Phylogenetic factors may contribute to segmental boundaries since meta-topology 5 mapped predominantly to allopallial structures (archipallium, paleopallium, and mesopallium), while meta-topologies 1–3 showed distinct neopallial patterns (parieto-occipital, frontal and temporal).^30^ In contrast, the parenchymal patterns in brain metastases corresponded to the border zones between the major cerebellar (meta-topology 1) and cerebral (meta-topology 2) arteries, consistent with previous descriptions of the metastatic tendency of origin in arterial watershed areas.^29^ Third, the meta-topologies in neuroepithelial tumours were strongly defined by distinct ventricular segments, while the ventricles did not contribute relevantly to the meta-topologies in brain metastases. In contrast to earlier beliefs, niches of neuroepithelial stem cells seem to persist in the adult brain serving as a source of continuous cell replenishment.^31,32^ Such specialized niches have been identified in the dentate gyrus and the subventricular zone of the lateral ventricles.^33-36^ It has been further hypothesized that glioblastoma, the most common neuroepithelial tumour, may arise from such stem cell niches.^37^ This might also apply to other or even all tumours of neuroepithelial origin.^38,39^ Stem cell niches of distinct localizations could explain the association between the automatically extracted meta-topologies and specific segments of the ventricular system. The meta-topologies identified in this study were characterized in particular by the frontal horn, atrium, temporal horn/dentate gyrus, and the lateral recess of the 4th ventricle. These locations are reminiscent of the periventricular niches harbouring stem cells and radial glia cells. Based on our patient-based findings, we thus hypothesize that specific radial ventriculo-cortical units are determined by their periventricular neuroepithelial stem cells and radial glial cells and potentially inform and shape the topological anatomy of neuroepithelial tumours.^9,40,41^

Patients with neuroepithelial tumours mapping to meta-topology 4 had a survival advantage after adjustment for tumour histology. This finding was confirmed within the group of patients with WHO Grade 4 gliomas. Meta-topology 4 showed a transpallial character, comparable with the other supratentorial meta-topologies in neuroepithelial tumours but lacked a specific parenchymal pattern. The unigyral character of meta-topology 4 explains its lack of a specific gyral pattern since topological analyses depend on the interrelationships between structures but not their mere involvement.^13^ Meta-topology 4 rather constitutes a less advanced tumour stage than a separate entity with the potential to progress to a specific topological pattern. Meta-topology 4 was determined by a focal tumour contact to the ventricle wall, distinguishing it from the other supratentorial meta-topologies associated with diffuse ventricle wall involvement. The contact pattern to the ventricular system was shown to be a relevant prognostic factor and constitutes a cornerstone of a previously proposed anatomical staging of neuroepithelial tumours.^9^

In brain metastases, melanoma and urogenital tract metastases contributed almost exclusively to the supratentorial meta-topology. The predominant supratentorial distribution is in accordance with previous studies suggesting a relative underrepresentation of melanoma metastases to the cerebellum.^42,43^ There was no evidence for a difference in survival between the infra- and supratentorial meta-topologies in brain metastases. The similarity may be explained by the fact that the presence of a specific brain tumour meta-topology is prognostically less critical than the stage and adjuvant treatment options of the underlying primary disease. The observation that meta-topologies do not offer a survival stratification in brain metastases is consistent with previous reports that the location of brain metastases is of minor overall prognostic significance.^44^

We introduce the concept of a data-driven analysis of topological classes in neuroepithelial tumours and brain metastases. The proposed machine learning framework offers an unsupervised approach to identifying latent brain tumour meta-topologies in a data-centric fashion.^45^ That is, the algorithm learns without labels directly from the raw anatomical profiles. Notably, information about the individual histology, molecular pathology, or clinical parameters is at no stage available to the algorithm. Yet, we show that coherent and plausible patterns can be analytically retrieved based on the anatomical structure alone.

Meta-topologies reflect segmental anatomical tumour behaviour with phylo- and ontogenetic rationalization. In the future, this may be relevant for both tumour classification and therapy. Complementing the current classification of neuroepithelial tumours, which is dominated by molecular and histological criteria, by a macroscopic, i.e. topological anatomical dimension, may enhance personalization in the management of brain tumour patients. In addition, surgical or radiotherapeutic interventions may be tailored to the patient-specific expression of meta-topologies in primary brain tumours. Based on the hypothesis that primary brain tumours, given their neuroepithelial nature, orient along defined radial ventriculo-cortical units, local therapy (surgery and radiotherapy) should potentially target the entire affected anatomical segment.

## Conclusions

We present a novel data-led framework capitalizing on non-negative matrix factorization to deconvolute generalizable topological patterns in brain tumours based solely on their anatomical profile. In neuroepithelial tumours, six meta-topologies with distinct parenchymal and ventricular compositions were identified. We were able to show that these meta-topologies map to distinct histopathologic, molecular and clinical findings, implying the existence of a linkage between the anatomical behaviour and biological signature of neuroepithelial tumours. This unsupervised anatomical categorization may complement the current molecular and histological classification of brain tumours by a macroscopic dimension. The gained insights through meta-topologies into the heterogeneous biology of tumour origin and spatial evolution offer new approaches to interpret research data, and potentially inform surgical and radiotherapeutic interventions tailored to the unique expression of meta-topologies.

## Supplementary Material

fcac336_Supplementary_Data

## Abbreviations

aRI =: adjusted Rand Index
NNMF =: non-negative matrix factorization
RE =: reconstruction error
