# Supplementary material for: Meta-topologies define distinct anatomical classes of brain tumours linked to histology and survival
Source: Brain Commun. 2022 Dec 22;5(1):fcac336. doi: 10.1093/braincomms/fcac336 (PMC9830987; doi:10.1093/braincomms/fcac336)
Supplement: fcac336_Supplementary_Data [file fcac336_supplementary_data.pdf]

Supplementary Material

Meta-topologies define distinct anatomical classes of brain tumors linked to histology and survival

Julius M. Kernbach[1,2,3*], Daniel Delev[1,2,3*], Georg Neuloh[2,3], Hans Clusmann[2,3], Danilo Bzdok[4,5], Simon B. Eickhoff [6,7], Victor E. Staartjes[8], Flavio Vasella[8], Michael Weller[9], Luca Regli[8], Carlo Serra[8], Niklaus Krayenbühl[8,10], Kevin Akeret[8]

1 Neurosurgical Artificial Intelligence Laboratory Aachen (NAILA), RWTH Aachen University Hospital, Aachen, Germany
2 Department of Neurosurgery, Faculty of Medicine, RWTH Aachen University, Aachen, Germany
3 Center for Integrated Oncology, Universities Aachen, Bonn, Cologne, Düsseldorf (CIO ABCD), Germany.
4 Department of Biomedical Engineering, McConnell Brain Imaging Centre, Montreal Neurological Institute, Faculty of Medicine, School of Computer Science, McGill University, Montreal, Canada
5 Mila - Quebec Artificial Intelligence Institute, Montreal, Canada
6 Institute of Neuroscience and Medicine (INM-7), Research Centre Jülich, Jülich, Germany.
7 Institute of Systems Neuroscience, Medical Faculty, Heinrich Heine University Düsseldorf, Düsseldorf, Germany.
8 Department of Neurosurgery, Clinical Neuroscience Center, University Hospital and University of Zurich, Zurich, Switzerland
9 Department of Neurology, Clinical Neuroscience Center, University Hospital and University of Zurich, Zurich, Switzerland
10 Division of Pediatric Neurosurgery, University Children's Hospital, Zurich, Switzerland
* shared

Corresponding author:
Kevin Akeret, MD PhD
Department of Neurosurgery, Clinical Neuroscience Center
University Hospital and University of Zurich
Frauenklinikstrasse 10
CH-8091 Zurich, Switzerland
Email: kevin.akeret@usz.ch
ORCID: 0000-0002-5946-4999

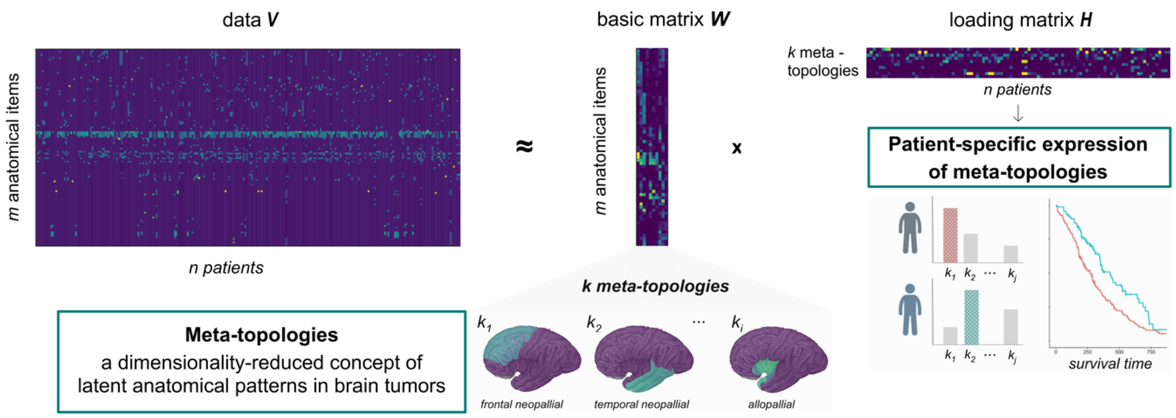

Supplementary Figure 1. Illustration of the NNMF factorization into meta-topologies

Non-negative matrix factorization (NNMF) was applied to the data *V* reflecting the 120 neuroanatomical items, with dimensions of *m* x *n* (*m* = number of anatomical items, *n* = number of patients). NNMF produces a factorization of the data V into a *basic matrix W* (meta-topologies) and a *loading matrix H*. The basic matrix W represents the low-dimensional latent anatomical patterns, that we termed meta-topologies. Each *k* meta-topology can be mapped to each corresponding anatomical item resulting in brain visualizations, e.g., k_1 as the frontal neopallial meta-topology. The loading matrix H summarizes the expression of the meta-topologies for every individual patient. These patient-specific expressions of tumor meta-topologies are then used as a biomarker and linked to differences in overall survival.

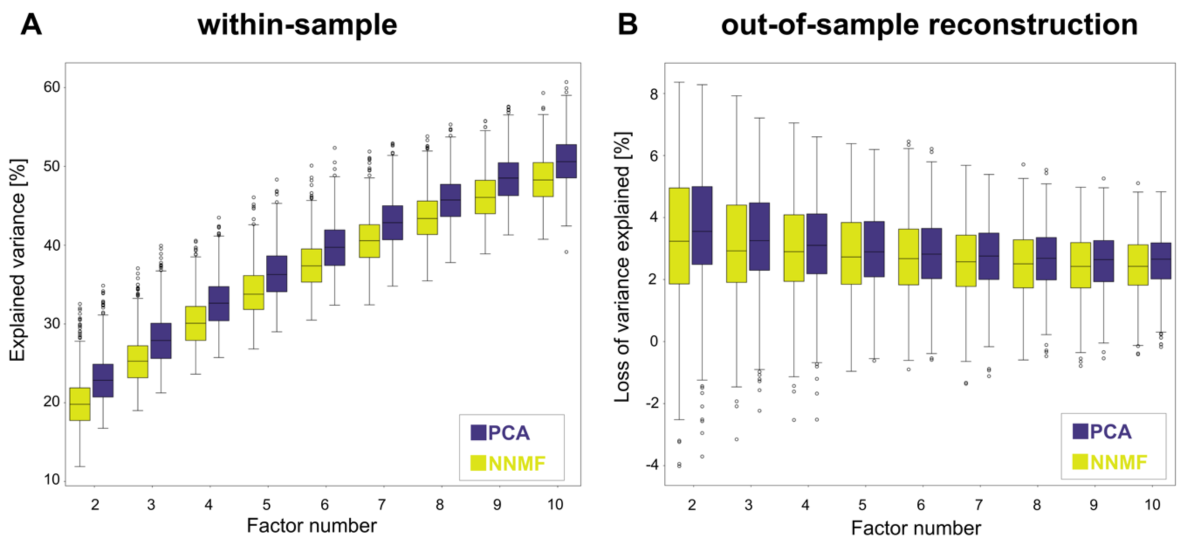

Supplementary Figure 2. Quantitative comparison of PCA and NNMF derived low-dimensional representations based on explained variance

A Within-sample explained variance (EV) for the basis matrix reconstructed by NNMF (magenta) and the PCA loadings (turquoise) in 1,000 bootstrap iterations. Higher variance explained by PCA compared to NNMF is not unexpected as NNMF applies regularizations and constraints which reduce the variance explained by the learned representation. **B** A higher loss of EV indicates worse generalizability. NNMF shows lower loss of EV, that is, showing a better generalization performance. In summary, PCA showed higher within-sample EV, but at the cost of lower interpretability. In turn, NNMF reached a slightly lower EV, but generalized better to new data with a lower loss of EV. In conclusion, based on a superior generalization combined with a more intuitive interpretability of a parts-based representation makes NNMF a more appropriate tool for capturing latent meta-topological patterns than PCA.

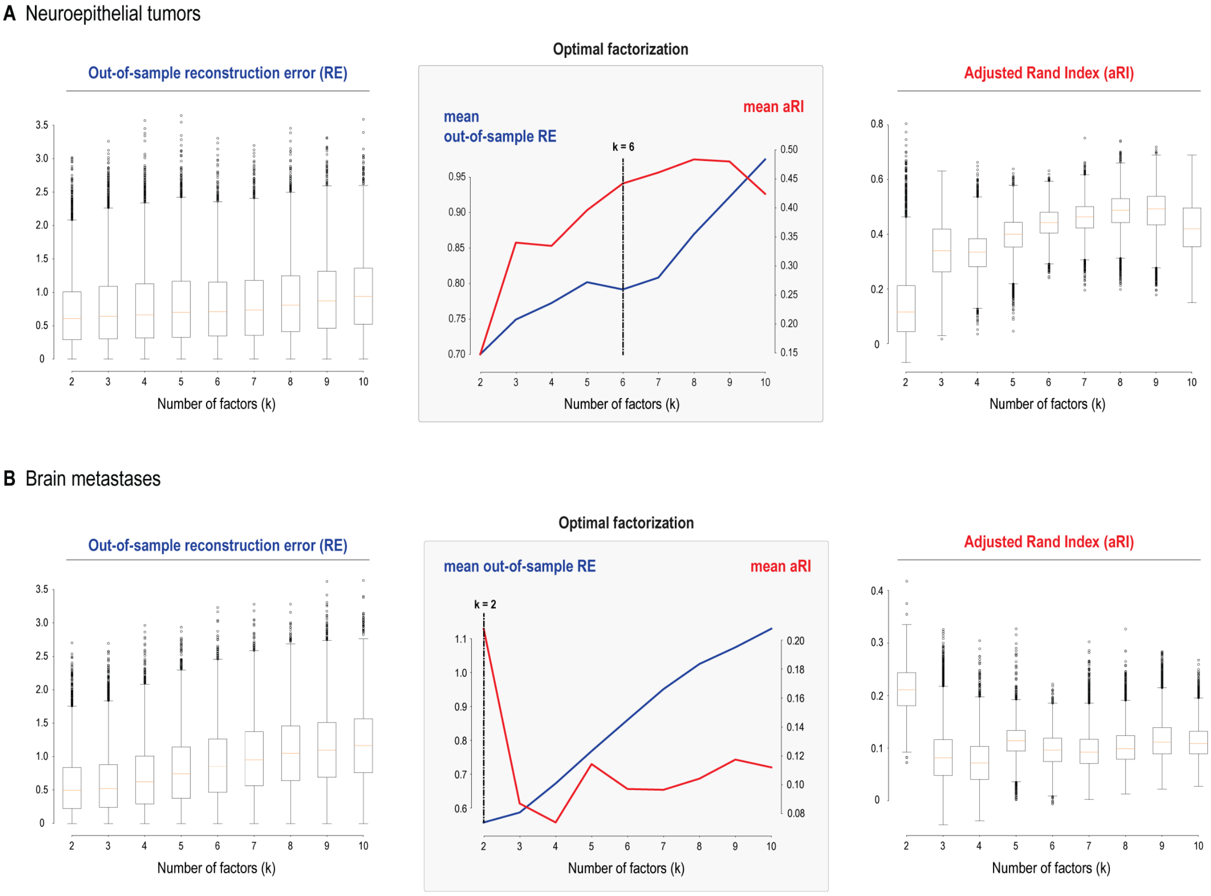

Supplementary Figure 3. Optimal factorizations in neuroepithelial tumors and brain metastases

Quantitative evaluation of different non-negative matrix factorization (NNMF) solutions with *k* number of factors assessed in 10,000 bootstrapped split-half analyses by the out-of-sample increase in the reconstruction error (RE) and the adjusted Rand Index (aRI). Lower values for the out-of-sample increase in RE indicate better generalizability. Higher values of aRI indicate higher stability. **A** The optimal factorization in neuroepithelial tumors resulted in *k*=6 meta-topologies. **B** The optimal factorization in brain metastases yielded *k*=2 meta-topologies.

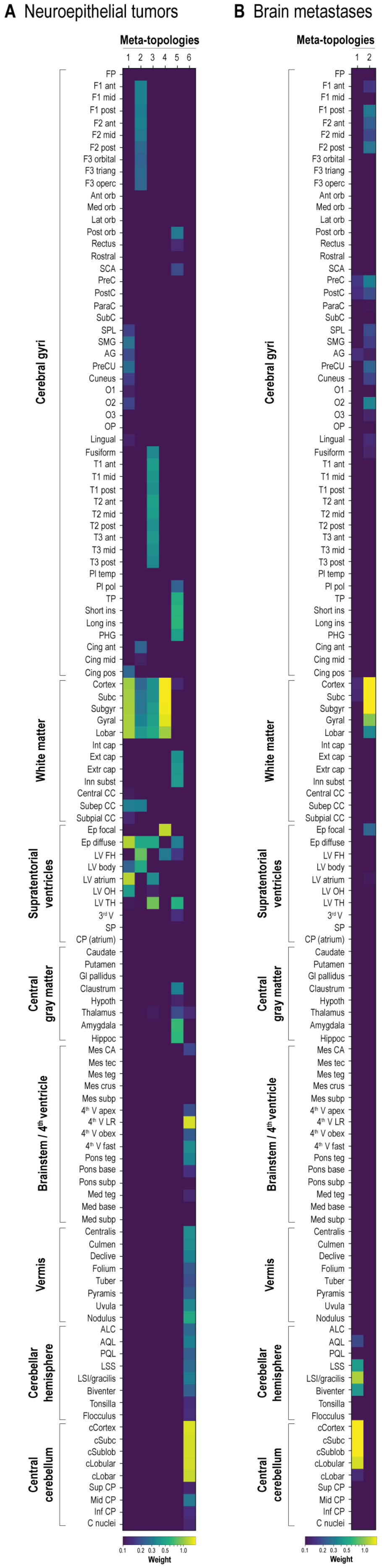
A Neuroepithelial tumors
B Brain metastases
Meta-topologies
Cerebral gyri
White matter
Supratentorial ventricles
Central gray matter
Brainstem / 4th ventricle
Vermis
Cerebellar hemisphere
Central cerebellum
Weight

Supplementary Figure 4. Detailed anatomy of meta-topologies in neuroepithelial tumors and brain metastases

Supplement to Figure 1 and 2: Meta-topologies in neuroepithelial tumors (**A**) and brain metastases (**B**) with their detailed underlying neuroanatomical distribution. *Abbreviations (in vertical descending order):* FP, frontal pole; F1, superior frontal gyrus; ant, anterior third; mid, middle third; post, posterior third; F2, middle frontal gyrus; F3 inferior frontal gyrus; Ant/Med/Lat/Post orb, anterior/medial/lateral/posterior orbital gyrus; SCA, subcallosal area; PreC, precentral gyrus; PostC, postcentral gyrus; ParaC, paracentral lobule; SubC, subcentral gyrus; SPL, superior parietal lobule; SMG, supramarginal gyrus; AG, angular gyrus; PreCU, precuneus; O1, superior occipital gyrus; O2, middle occipital gyrus; O3, inferior occipital gyrus; OP, occipital pole; T1, superior temporal gyrus; T2, middle temporal gyrus; T3, inferior temporal gyrus; Pl temp, planum temporale; Pl pol, planum polare; TP, temporal pole; ins, insular gyri; PHG, parahippocampal gyrus; Cingulate, cingulate gyrus; SubC, subcortical white matter sector; Subgyr, subgyral white matter sector; Gyral, gyral white matter sector; Lobar, lobar white matter sector; Int cap, internal capsule; Ext cap, external capsule; Extr cap, extreme capsule; Inn subst, innominate substance; CC, corpus callosum; Subep, subependymal; Ep, ependyma; LV, lateral ventricle; FH, frontal horn; OH, occipital horn; 3rd V, third ventricle; SP, septum pellucidum; CP, choroid plexus; Gl, globus; Hypoth, hypothalamus; Hippoc, hippocampus; Mes, mesencephalon; CA, cerebral aqueduct (of Sylvius); tec, tectum; teg, tegmentum; subp, subpial; 4th V, fourth ventricle; LR, lateral recess; fast, fastigium; ALC, ala lobuli centralis; AQL, anterior quadrangular lobule; PQL, posterior quadrangular lobule; LSS, superior semilunar lobule; LSI, inferior semilunar lobule; cCortex, cerebellar cortex; sSubc, cerebellar subcortical white matter sector; cSublob, cerebellar sublobular white matter sector; cLobular, cerebellar lobular white matter sector; cLobar, cerebellar lobar white matter sector; CP, cerebellar peduncle; C nuclei, cerebellar nuclei.

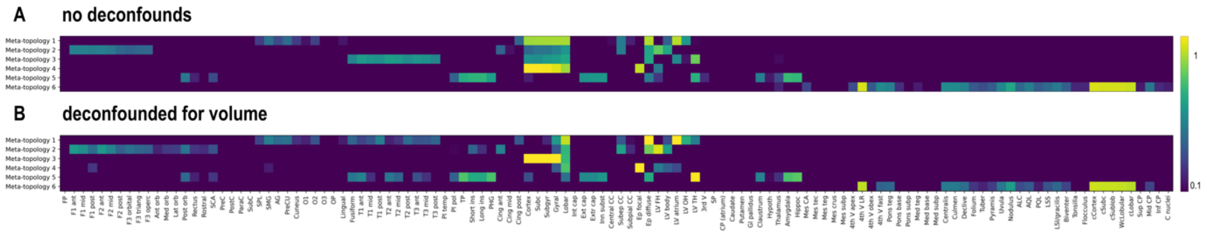

Supplementary Figure 5. NNMF deconfounded for lesion volume

The original NNMF clustering in neuroepithelial tumors (A) compared to a NNMF clustering deconfounded for lesion volume (B). Participants that did not have volumetric measurements were excluded (n=102). The characteristic combination of topographic items remains similar for most meta-topologies. One part-based representation of superficial-towards-deep white matter sectors emerges as new individual factor (B, meta-topology 3), and is hence less represented in the other meta-topologies (previously meta-topologies 1-4). Additionally, the part-representation of the temporal areas (previously in meta-topology 3) is now combined in meta-topology 1.

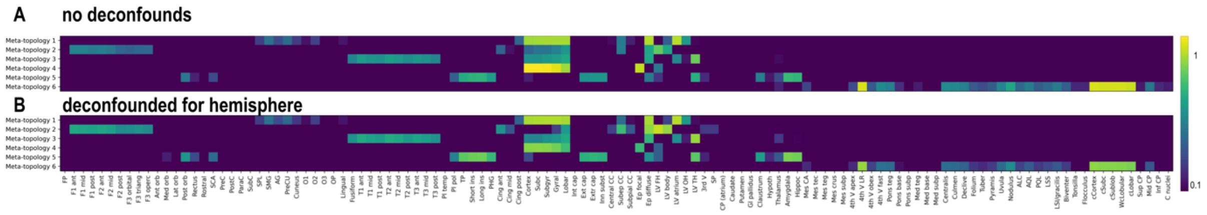

Supplementary Figure 6. NNMF deconfounded for hemispheric lateralization

The original NNMF clustering in neuroepithelial tumors (A) compared to a new NNMF clustering deconfounded for hemispheric lateralization (B). Adding hemispheric lateralization as a deconfounding variable results in a virtually identical NNMF clustering.

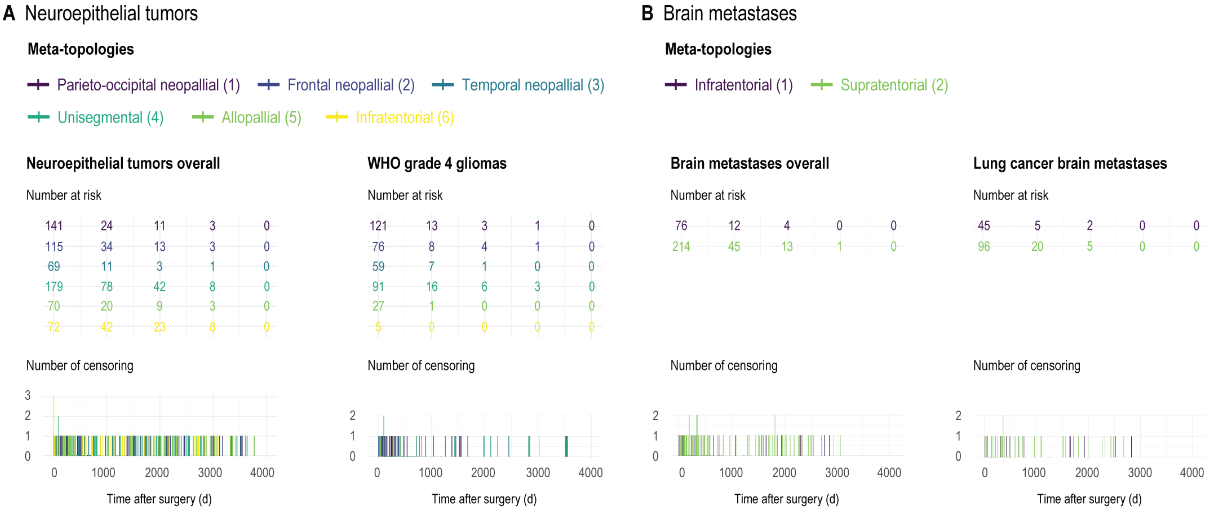

Supplementary Figure 7. Risk tables and censoring events for the meta-topology-specific survival analyses

Supplement to the Kaplan-Meier curves shown in Figures 4 and 5 providing the risk for an event over time (number at risk) and the number of censoring events over time (number of censoring) stratified by the corresponding meta-topology of highest relevance. **A.** Neuroepithelial tumors overall (left) and WHO grade 4 gliomas (right). *Abbreviations:* d, days. **B.** Brain metastases overall (left) and lung cancer brain metastases (right).

Supplementary Table 1. Cohort characteristics

The demographic, histopathologic, and clinical characteristics of the study cohort. *Abbreviations:* DT, developmental tumors; EP, ependymoma; g2G, WHO grade 2 gliomas; g3G, WHO grade 3 gliomas; g4G, WHO grade 4 gliomas; GIT, gastrointestinal tract (mouth, tonsil, parotid, esophagus, stomach, gallbladder, pancreas, colorectal cancer); MB, medulloblastoma; Misc., miscellaneous (cancer of unknown primary, adrenal, leukemia, sarcoma, mesothelial, thyroid); PA, pilocytic astrocytoma; UGT, urogenital tract (kidney, bladder; ovary, tube, uterus; testes, prostate).

*based on immunohistochemistry or PCR

Neuroepithelial tumors

	**Overall**	**g4G**	**g3G**	**g2G**	**DT**	**EP**	**PA**	**MB**
**n**	646	379	105	50	19	26	30	15
**Sex (male)**	402 (62.2)	244 (64.4)	63 (60.0)	31 (62.0)	12 (63.2)	14 (53.8)	21 (70.0)	9 (60.0)
**Age (years)**	50.1 (21.6)	60.8 (14.2)	46.4 (15.7)	43.1 (16.1)	20.4 (15.7)	24.6 (21.3)	11.7 (10.4)	16.6 (14.3)
**Karnofsky Performance Status**	80.0 (12.6)	76.9 (13.2)	84.1 (10.0)	86.7 (9.9)	87.6 (8.3)	86.7 (11.4)	82.9 (9.2)	83.0 (6.7)
**Modified Rankin Scale**	1.7 (1.0)	1.9 (1.0)	1.4 (0.7)	1.2 (0.7)	1.1 (0.6)	1.3 (0.9)	1.5 (0.7)	1.5 (0.5)
**Resection (vs. biopsy)**	510 (78.9)	285 (75.2)	81 (77.1)	37 (74.0)	19 (100.0)	24 (92.3)	30 (100.0)	14 (93.3)
**Chemotherapy**	428 (69.1)	269 (73.7)	89 (86.4)	35 (71.4)	0 (0.0)	13 (59.1)	2 (7.1)	14 (93.3)
**Radiotherapy**	473 (76.2)	311 (85.0)	91 (88.3)	29 (58.0)	2 (10.5)	17 (77.3)	0 (0.0)	15 (100.0)
**MIB1**	25.0 (20.6)	32.5 (19.2)	18.7 (17.4)	6.7 (4.8)	3.4 (4.4)	25.0 (24.4)	3.8 (3.6)	38.3 (26.5)
**1p19q co-deletion**	-	-	19 (23.8)	21 (61.8)	-	-	-	-
**IDH1 mutation***	-	10 (3.4)	45 (51.1)	32 (72.7)	-	-	-	-
**MGMT promoter methylation**	-	82 (38.0)	9 (45.0)	4 (57.1)	-	-	-	-

Metastases

	**Overall**	**Lung**	**Skin**	**GIT**	**Breast**	**UGT**	**Misc.**
**n**	290	141	44	37	27	24	17
**Sex (male)**	149 (51.4)	77 (54.6)	24 (54.5)	24 (64.9)	1 (3.7)	16 (66.7)	7 (41.2)
**Age (years)**	60.7 (11.9)	61.4 (9.9)	58.4 (13.6)	65.6 (10.3)	58.4 (12.5)	56.2 (18.3)	60.4 (10.3)
**Karnofsky Performance Status**	76.9 (11.6)	77.7 (11.0)	79.8 (11.1)	74.1 (10.4)	77.8 (11.5)	71.2 (15.1)	75.9 (12.3)
**Modified Rankin Scale**	2.0 (0.9)	1.9 (0.8)	1.8 (1.0)	2.2 (0.8)	1.9 (0.8)	2.4 (1.0)	1.9 (1.0)
**Resection (vs. biopsy)**	277 (95.5)	136 (96.5)	43 (97.7)	36 (97.3)	27 (100.0)	23 (95.8)	12 (70.6)
**Chemotherapy**	175 (62.3)	94 (69.1)	34 (77.3)	12 (33.3)	17 (65.4)	10 (41.7)	8 (53.3)
**Radiotherapy**	255 (89.2)	128 (92.1)	42 (95.5)	31 (83.8)	25 (92.6)	18 (75.0)	11 (73.3)

Supplementary Table 2. Differential meta-topological anatomy in neuroepithelial tumors

Top ten anatomical items that define each meta-topology in neuroepithelial tumors.

Factor	Anatomical item	Weight	Factor	Anatomical item	Weight
**1**	Lateral ventricle – atrium	**1.19**	**4**	Cerebral cortex	**1.58**
	Ventricle wall – diffuse	**1.17**		Cerebral subcortical white matter	**1.57**
	Cerebral gyral white matter	**1.11**		Cerebral subgyral white matter	**1.54**
	Cerebral lobar white matter	**1.11**		Cerebral gyral white matter	**1.41**
	Cerebral subgyral white matter	**1.10**		Ventricle wall – focal	**1.25**
	Cerebral subcortical white matter	**1.09**		Cerebral lobar white matter	**1.07**
	Cerebral cortex	**1.07**		Lateral ventricle – frontal horn	**0.30**
	Lateral ventricle – occipital horn	**0.45**		Subcentral gyrus	**0.09**
	Subependymal corpus callosum	**0.32**		Short insular gyri	**0.06**
	Supramarginal gyrus	**0.28**		Medial orbital gyrus	**0.06**
**2**	Lateral ventricle – frontal horn	**0.82**	**5**	Amygdala	**0.65**
	Lateral ventricle – body	**0.52**		Hippocampus	**0.62**
	Ventricle wall – diffuse	**0.52**		Long insular gyri	**0.61**
	Cerebral lobar white matter	**0.43**		Short insular gyri	**0.60**
	Superior frontal gyrus – middle	**0.34**		Lateral ventricle – temporal horn	**0.57**
	Superior frontal gyrus – anterior	**0.34**		Temporal pole	**0.56**
	Cerebral gyral white matter	**0.34**		Parahippocampal gyrus	**0.47**
	Middle frontal gyrus – anterior	**0.33**		Innominate substance	**0.44**
	Subependymal corpus callosum	**0.32**		Extreme capsule	**0.43**
	Superior frontal gyrus – posterior	**0.32**		External capsule	**0.41**
**3**	Lateral ventricle – temporal horn	**0.86**	**6**	Cerebellar cortex	**1.37**
	Ventricle wall – diffuse	**0.53**		4th ventricle – lateral recess	**1.35**
	Cerebral lobar white matter	**0.52**		Cerebellar sublobular white matter	**1.34**
	Middle temporal gyrus – anterior	**0.47**		Cerebellar subcortical white matter	**1.34**
	Cerebral gyral white matter	**0.47**		Cerebellar lobular white matter	**1.33**
	Superior temporal gyrus – anterior	**0.46**		Cerebellar lobar white matter	**1.27**
	Middle temporal gyrus – middle	**0.45**		Vermis – nodulus	**0.52**
	Cerebral subgyral white matter	**0.44**		Vermis – centralis	**0.38**
	Inferior temporal gyrus – anterior	**0.42**		4th ventricle – fastigium	**0.38**
	Superior temporal gyrus – middle	**0.42**		Vermis – culmen	**0.38**

Supplementary Table 3. Gyrality of neuroepithelial tumor meta-topologies

The gyral character of each meta-topology, i.e., the number (*n*) and proportion (*%*) of tumors without gyral involvement (none), those involving one gyrus (unigyral), and tumors involving multiple gyri (multigyral).

		Meta-topology					
	**Overall**	**1**	**2**	**3**	**4**	**5**	**6**
**n**	646	141	115	69	179	70	72
**Gyrality (%)**							
**none**	119 (18.4)	6 (4.3)	17 (14.8)	3 (4.3)	0 (0.0)	22 (31.4)	71 (98.6)
**unigyral**	285 (44.1)	70 (49.6)	32 (27.8)	27 (39.1)	147 (82.1)	9 (12.9)	0 (0.0)
**multigyral**	242 (37.5)	65 (46.1)	66 (57.4)	39 (56.5)	32 (17.9)	39 (55.7)	1 (1.4)

Supplementary Table 4. Differential meta-topological anatomy in brain metastases

Top ten anatomical items that define meta-topologies 1 and 2 in brain metastases.

Factor	Anatomical item	Weight
**1**	Cerebellar cortex	**2.18**
	Cerebellar subcortical white matter	**2.17**
	Cerebellar sublobular white matter	**1.94**
	Cerebellar lobular white matter	**1.49**
	Inferior semilunar / gracile lobule	**1.24**
	Superior semilunar lobule	**0.47**
	Biventer lobule	**0.45**
	Anterior quadrangular lobule	**0.19**
	Precentral gyrus	**0.16**
	Postcentral gyrus	**0.15**
**2**	Cerebral subcortical white matter	**2.83**
	Cerebral cortex	**2.83**
	Cerebral subgyral white matter	**2.30**
	Cerebral gyral white matter	**1.03**
	Cerebral lobar white matter	**0.37**
	Middle occipital gyrus	**0.36**
	Precentral gyrus	**0.34**
	Superior frontal gyrus – posterior	**0.32**
	Middle frontal gyrus – posterior	**0.30**
	Ventricle wall – focal	**0.26**

Supplementary Table 5. Pairwise comparison of survival curves stratified by meta-topologies in neuroepithelial tumors overall and WHO grade 4 gliomas

Evidence for a difference between the meta-topology-stratified survival curves in (**A**) neuroepithelial tumors overall and in (**B**) WHO grade 4 gliomas according to pairwise comparison with the log-rank test and Bonferoni-Holm correction for multiple testing. The p-value is based on the log-rank test (Chi-square test statistic χ^2). ****very strong evidence (p < 0.001), **strong evidence (p < 0.01), *evidence (p < 0.05), +weak evidence (p < 0.1).*

Meta-topology	1	2	3	4	5
			**(A) Neuroepithelial tumors overall**		
**2**	p = 0.033* (χ^2 = 4.57)	NA	NA	NA	NA
**3**	p = 0.68 (χ^2 = 0.17)	p = 0.023* (χ^2 = 5.20)	NA	NA	NA
**4**	p < 0.0001*** (χ^2 = 38.8)	p = 0.0045** (χ^2 = 8.06)	p <0.0001*** (χ^2 = 35.1)	NA	NA
**5**	p = 0.0084** (χ^2 = 6.93)	p = 0.48 (χ^2 = 0.51)	p = 0.0055** (χ^2 = 7.71)	p = 0.096+ (χ^2 = 2.77)	NA
**6**	p < 0.0001*** (χ^2 = 59.4)	p < 0.0001*** (χ^2 = 31.7)	p < 0.0001*** (χ^2 = 59.3)	p < 0.0001*** (χ^2 = 19.5)	p < 0.0001*** (χ^2 = 22.6)
			**(B) WHO grade 4 gliomas**		
**2**	p = 0.39 (χ^2 = 0.75)	NA	NA	NA	NA
**3**	p = 0.81 (χ^2 = 0.060)	p = 0.71 (χ^2 = 0.14)	NA	NA	NA
**4**	p = 0.0051** (χ^2 = 7.84)	p = 0.0010** (χ^2 = 10.8)	p = 0.0079** (χ^2 = 7.05)	NA	NA
**5**	P = 0.17 (χ^2 = 1.85)	p = 0.66 (χ^2 = 0.19)	p = 0.21 (χ^2 = 1.56)	p = 0.0005*** (χ^2 = 12.1)	NA
**6**	p = 0.26 (χ^2 = 1.29)	p = 0.60 (χ^2 = 0.27)	p = 0.20 (χ^2 = 1.67)	p = 0.025* (χ^2 = 5.05)	p = 0.47 (χ^2 = 0.53)
